# Malaria and immunity during pregnancy and postpartum: a tale of two species

**DOI:** 10.1017/S0031182015000074

**Published:** 2015-03-03

**Authors:** A. R. D. McLEAN, R. ATAIDE, J. A. SIMPSON, J. G. BEESON, F. J. I. FOWKES

**Affiliations:** 1 Macfarlane Burnet Institute of Medical Research, 85 Commercial Road, Melbourne, Victoria 3004, Australia; 2 Centre for Epidemiology and Biostatistics, Melbourne School of Population and Global Health, The University of Melbourne, Melbourne, Victoria, Australia; 3 Department of Microbiology, Monash University, Melbourne, Victoria, Australia; 4 Department of Medicine, University of Melbourne, Melbourne, Victoria, Australia; 5 Department of Epidemiology and Preventive Medicine and Department of Infectious Diseases, Monash University, Commercial Road, Melbourne, Victoria 3004, Australia

**Keywords:** malaria, *Plasmodium vivax*, falciparum, pregnancy, postpartum, immunity, epidemiology

## Abstract

It is well established that pregnant women are at an increased risk of *Plasmodium falciparum* infection when compared to non-pregnant individuals and limited epidemiological data suggest *Plasmodium vivax* risk also increases with pregnancy. The risk of *P. falciparum* declines with successive pregnancies due to the acquisition of immunity to pregnancy-specific *P. falciparum* variants. However, despite similar declines in *P. vivax* risk with successive pregnancies, there is a paucity of evidence *P. vivax*-specific immunity. Cross-species immunity, as well as immunological and physiological changes that occur during pregnancy may influence the susceptibility to both *P. vivax* and *P. falciparum*. The period following delivery, the postpartum period, is relatively understudied and available epidemiological data suggests that it may also be a period of increased risk of infection to *Plasmodium* spp. Here we review the literature and directly compare and contrast the epidemiology, clinical pathogenesis and immunological features of *P. vivax* and *P. falciparum* in pregnancy, with a particular focus on studies performed in areas co-endemic for both species. Furthermore, we review the intriguing epidemiology literature of both *P. falciparum* and *P. vivax* postpartum and relate observations to the growing literature pertaining to malaria immunology in the postpartum period.

## INTRODUCTION

Malaria is a mosquito-borne infectious disease caused by the parasite *Plasmodium*, of which six species can infect humans; *Plasmodium falciparum, Plasmodium vivax, Plasmodium ovale curtisi, Plasmodium ovale wallikeri, Plasmodium malariae* and *Plasmodium knowlesi*. Of these, *P. falciparum* and *P. vivax* are the predominant species with an estimated 182·2 million clinical cases of *P. falciparum* malaria, 15·8 million clinical cases of *P. vivax* malaria and 584 000 deaths attributable to malaria every year (WHO, 2014). The greatest burden of disease is seen in young children and pregnant women. *P. falciparum* is responsible for the vast majority of global morbidity and mortality (WHO, 2014). It is estimated that over 125 million pregnancies are at risk of malaria, 32 million are at risk of *P. falciparum*, 40 million are at risk of *P. vivax* and 53 million are at risk of both species) (Dellicour *et al.* 2010). Women who acquire a *Plasmodium* spp. infection during pregnancy commonly experience negative maternal and birth outcomes such as anaemia, low birth weight and preterm birth with an estimated 75 000–200 000 infant deaths annually attributable to malaria in pregnancy (Steketee *et al.* 2001). Malaria in the period following pregnancy, the postpartum period, is also of public health importance. Malaria was the leading indirect cause of death in postpartum women in a study in Zambia (Vallely *et al.* 2005) and the second highest cause of postpartum death in a study in India (Barnett *et al.* 2008).

In malaria endemic areas, individuals develop naturally acquired immunity to both *P. falciparum* and *P. vivax* after repeated infections. This immunity does not generally protect against infection *per se*, but protects against the development of high parasite densities and clinical symptoms (reviewed in Langhorne *et al.* 2008). Despite acquiring a degree of protective immunity prior to pregnancy, pregnant women are typically more susceptible to *P. falciparum* and *P. vivax.* Broad hormonal and immunological changes that occur during pregnancy are likely to play a role, with a general shift from cell-mediated immunity toward humoral immunity (Jamieson *et al.* 2006; Robinson and Klein, 2012). In the case of *P. falciparum* the increased susceptibility has been largely attributed to the lack of immunity to pregnancy-specific isolates that sequester in the placenta (well documented and extensively reviewed elsewhere, e.g. (Desai *et al.* 2007; Duffy, 2007; Hviid and Salanti, 2007; Rogerson, 2010; Umbers *et al.* 2011)). The ability of *P. vivax* to bind and sequester in the placenta, its role in pathogenesis and the role of immunity against this process are debated (Mayor *et al.* 2012*a*). Importantly, *P. vivax* possesses the ability to form hypnozoites in the liver, a dormant stage which can lead to relapses of blood-stage infections (Krotoski *et al.* 1982; Krotoski, 1985). The immunological mechanisms that mitigate *P. vivax* in pregnancy are unclear, as is the effect of an altered immunological state during pregnancy on the risk of relapse.

The rate at which a woman returns to a normal immunological state after pregnancy, and how this affects malaria risk postpartum has not been well characterized. There is increasing evidence for the altered susceptibility to *P. falciparum* and *P. vivax* postpartum (Boel *et al.* 2012) and a growing literature investigating the immune response to malaria in the postpartum period which may account for observed epidemiological patterns. In this review we highlight the similarities and differences of *P. vivax* and *P. falciparum* infection during pregnancy and the postpartum period with respect to epidemiology, clinical pathogenesis and immunology.

### Plasmodium falciparum and P. vivax risk in pregnancy

Numerous studies have demonstrated that pregnant women are at increased risk of *P. falciparum* infection and experience higher parasite densities and rates of clinical malaria when compared to non-pregnant women (reviewed in Desai *et al.* 2007). We therefore reviewed the *P. vivax* literature in addition to studies investigating *P. vivax* and *P. falciparum* in co-endemic areas. Few studies have investigated the risk of *P. vivax* infection during pregnancy and available data is conflicting (Table 1). An increased risk of *P. vivax* infection (Singh *et al.* 1995, 1999) and increased density of *P. vivax* infections (Campbell *et al.* 1980; Singh *et al.* 1999) have been observed in pregnant compared to non-pregnant women from El Salvador and India (Table 1). An increased multiplicity of *P. vivax* infections during pregnancy in Thailand has also been observed (Thanapongpichat *et al.* 2013) though no difference was observed in Brazil (Marin-Menendez *et al.* 2013). Other studies have failed to detect substantial differences in *P. vivax* risk between pregnant women and non-pregnant women (Campbell *et al.* 1980; Parekh *et al.* 2007) (Table 1). Furthermore, a study in Brazil found an increased frequency of *P. falciparum* relative to *P. vivax* infections in pregnant compared to non-pregnant women (Martinez-Espinosa *et al.* 2004). However, another study from the same Brazilian population failed to replicate these findings (Almeida *et al.* 2010) and to further complicate matters, a study in Indonesia found an increased frequency of *P. vivax* relative to *P. falciparum* in pregnancy (Barcus *et al.* 2007) (Table 1). The available evidence is somewhat conflicting but together suggests that there is an increased risk of *P. vivax* infection during pregnancy compared to non-pregnancy, albeit a smaller increased risk than that observed in regards to pregnancy and *P. falciparum* infection.

**Table 1.** *Plasmodium vivax* risk in pregnancy compared to non-pregnant women and comparisons with *P. falciparum* risk in co-endemic areas

Study	Country	*Plasmodium vivax*	Magnitude (95% CI)	*Plasmodium falciparum*	Magnitude (95% CI)
Risk/odds of infection					
Campbell *et al.* (1980)	El Salvador	Similar	RR = 1·13 (0·83, 1·53)^a^	Increased	RR = 1·23 (0·93, 1·63)^a^
Singh *et al.* (1995)^b^	India	Increased	OR = 1·30 (0·82, 2·06)^a^	Increased	OR = 2·34 (1·63, 3·37)^a^
Singh *et al.* (1999)^b^	India	Increased	OR = 3·36 (2·28, 5·07)^a^	Increased	OR = 2·11 (1·66, 2·68)^a^
Parekh *et al.* (2007)	Peru	Similar	RR = 0·92 (0·52, 1·64)	Increased	RR = 2·28 (1·32, 3·95)
Multiplicity of infection					
Marin-Menendez *et al.* (2013)	Brazil	Similar MOI	1·17 MOI *vs* 1·17 MOI	N/A	N/A
Thanapongpichat *et al.* (2013)	Thailand	Increased MOI	2·03 MOI *vs* 1·65 MOI	N/A	N/A
Species-specific parasite mean density					
(Campbell *et al.* 1980)	El Salvador	Increased	MD = +1615/mm^3^^a^	Increased	MD = +3093/mm^3^^a^
(Singh *et al.* 1999)^b^	India	Increased	MD = +11369/mm^3^^a^	Increased	MD = +8265/mm^3^^a^
Species ratio		Pv:Pf		Pf:Pv	
Martinez-Espinosa *et al.* (2004)†	Brazil	Decreased	2·3:1 *vs* 5·6:1	Increased	1:2·3 *vs* 1:5·6
Barcus *et al.* (2007)^c^	Indonesia	Increased	1:2·6 *vs* 1:3·7	Decreased	2·6:1 *vs* 3·7:1
Almeida *et al.* (2010)^b^	Brazil	Similar	5·8:1 *vs* 5·5:1	Similar	1:5·8 *vs* 1:5·5

NB – ratios within 0·2 of 1 were considered similar to 1. Abbreviations: MD, mean difference; OR, odds ratio; RR, risk ratio; N/A, not available; MOI, multiplicity of infections. All measures of association are unadjusted unless otherwise specified.

a Calculated from data in paper.

b Women in study restricted to those with history of fever.

c Women with slide-confirmed diagnoses of malaria.

The clinical consequences of *Plasmodium* infection occur during the blood-stage of infection and are exacerbated by high densities of the blood-stage parasite. *P. falciparum* invades all erythrocytes, whereas *P. vivax* selectively invades young erythrocytes (reticulocytes), and thus *P. vivax* parasitaemia is typically lower than *P. falciparum* parasitaemia (Collins and Jeffery, 1999*a*, *b*; Simpson *et al.* 1999; Collins *et al.* 2004). *P. vivax* has a lower pyrogenic threshold compared to *P. falciparum,* provoking a stronger inflammatory response for a given level of parasitaemia (Ross and Thomson, 1910; Luxemburger *et al.* 1996; Hemmer *et al.* 2006; Yeo *et al.* 2010). However, *P. vivax* infections less frequently progress to severe disease compared to *P. falciparum* infections, which can result in cerebral malaria, metabolic acidosis, respiratory distress and severe anaemia. *P. vivax* can lead to severe clinical symptoms such as severe anaemia, respiratory distress and thrombocytopenia (reviewed in Anstey *et al.* 2012).

Few studies have investigated the relative severity of *P. falciparum* compared to *P. vivax* during pregnancy on maternal outcomes in co-endemic populations. Most studies show that *P. falciparum* is associated with more severe maternal and birth outcomes (Tables 2 and 3). Studies in Thailand, India and Indonesia have demonstrated that pregnant women infected with *P. falciparum* have increased severity and odds of anaemia compared to those infected with *P. vivax* (Nair and Nair, 1993; Nosten *et al.* 1999; Singh *et al.* 1999; Poespoprodjo *et al.* 2008). Interestingly, a study in Thailand has indicated a potential interaction in disease severity between the two species demonstrating a protective effect of *P. vivax* infection against severity and number of *P. falciparum* episodes during pregnancy (Luxemburger *et al.* 1997; Nosten *et al.* 1999). Both *P. falciparum* and *P. vivax* infections during pregnancy are associated with detrimental birth outcomes such as low birth weight, preterm delivery and miscarriage (Table 3). Studies conducted in Thailand, India, Colombia and Indonesia have tended to find a greater reduction in birth weight and greater increase in the risk of preterm delivery amongst pregnant women with *P. falciparum* infections compared to *P. vivax* infections in pregnancy (Nair and Nair, 1993; Nosten *et al.* 1999; Singh *et al.* 1999; McGready *et al.* 2004; Poespoprodjo *et al.* 2008; Tobon-Castano *et al.* 2011) (Table 3). A study in India showed reduced odds of foetal loss in *P. vivax* compared to *P. falciparum* infections (Nair and Nair, 1993), whereas studies in Thailand which have specifically examined miscarriage found similar odds in *P. falciparum* and *P. vivax* infections (McGready *et al.* 2012). Taken together the above findings suggest that some of the underlying mechanisms by which the two species mediate negative birth outcomes are independent.

**Table 2.** Adverse maternal outcomes due to *P. vivax* infection in pregnancy compared to non-infected pregnant women and comparisons with *P. falciparum* risk in co-endemic areas

Study	Country	*Plasmodium vivax*	Magnitude (95% CI)	*Plasmodium falciparum*	Magnitude (95% CI)
Risk/odds of anaemia					
Nair and Nair (1993)^a^	India	Increased^b^	OR = 1·24 (0·53, 2·81)^c^	Increased^b^	OR = 3·22 (1·53, 6·78)^c^
Nosten *et al.* (1999)	Thailand	Increased^d^	OR = 1·91 (1·42, 2·56)^e^	N/A	N/A
Dreyfuss *et al.* (2000)	Nepal	Increased^f^	OR = 2·24 (0·91, 5·52)^g^	N/A	N/A
Mean difference in haemoglobin					
Singh *et al.* (1999)^a^	India	Decreased	MD = −0·98 g dl^−1^ (−1·11, −0·85)^c^	Decreased	MD=−3·61 g dl^−1^ (−3·76, −3·46)^c^
Poespoprodjo *et al.* (2008)	Indonesia	Decreased	MD = −0·4 g dl^−1^ (0·7, −0·1)	Decreased	MD=−1·1 g dl^−1^ (−1·4, −1·0)
Yasnot *et al.* (2013)	Colombia	Similar	MD = −0·1 g dl^−1^ (−1·29, 1·09)	N/A	N/A
Machado Filho *et al.* (2014)	Brazil	Decreased	MD = 1·2 g dl^−1^ (−1·79, −0·61)	N/A	N/A

NB – ratios within 0.2 of 1 and mean differences of less than 0.2 g dl^−1^ were considered similar. All measures of association are unadjusted unless otherwise specified. Abbreviations: MD, mean difference; OR, odds ratio; N/A, not available.

a Women in study restricted to those with history of fever.

b Anaemia defined as <8 hb g dl^−1^.

c Calculated from data in paper.

d Anaemia defined clinically or by haematocrit <30%.

e Adjusted for age, location, gestational age at first visit, compliance to attendance at the antenatal clinic.

f Anaemia defined as <11 hb g dl^−1^.

g Adjusted for hookworm infection, vitamin A deficiency and trimester of pregnancy.

**Table 3.** Adverse birth outcomes due to *P. vivax* infection in pregnancy compared to non-infected pregnant women and comparisons with *P. falciparum* risk in co-endemic areas

Study	Country	*Plasmodium vivax*	Magnitude (95% CI)	*Plasmodium falciparum*	Magnitude (95% CI)
Risk/odds of low birth weight					
McGready *et al.* (2004)^a^	Thailand	Decreased	OR = 0·31 (0·01, 4·21)^b^	Increased	OR = 1·76 (0·44, 10·18)^b^
Poespoprodjo *et al.* (2008)	Indonesia	Increased	OR = 1·9 (1·2, 3·1)	Increased	OR = 1·9 (1·4, 2·7)
Tobon-Castano *et al.* (2011)	Colombia	Increased	RR = 1·26 (0·80, 1·98)^b^	Increased	RR = 2·12 (1·24, 3·60)^b^
Mean difference in birth weight					
Nosten *et al.* (1991)	Thailand	Similar	MD = −49 g^b^	Decreased	MD = −128 g^b^
Nair and Nair (1993)^c^	India	Decreased	MD = −390 g^b^	Decreased	MD = −780 g^b^
Nosten *et al.* (1999)	Thailand	Decreased^d^	MD = −107 g (−154, 61)^b^	N/A	N/A
Singh *et al.* (1999)^c^	India	Decreased	MD = −310 g (−356, −264)^b^	Decreased	MD = −380 g (−425, −335)^b^
McGready *et al.* (2004)^a^	Thailand	Similar	MD = −10 g (−182, 162)^b^	Decreased	MD = −80 g (−288, 128)^b^
Poespoprodjo *et al.* (2008)	Indonesia	Decreased	MD = −108 g (−199, −18)	Decreased	MD = −192 g (−265, −119)
Arango *et al.* (2013)	Colombia	Decreased	MD = −525 g (−780, −270)^b^	Decreased	MD = −278 g (−771, 215)^b^
Yasnot *et al.* (2013)	Colombia	Decreased	MD = −215 g (−539, 109)^b^	N/A	N/A
Machado Filho *et al.* (2014)	Brazil	Decreased	MD = −434 g (−742, −127)^b^	N/A	N/A
Risk/odds of preterm delivery					
Nair and Nair (1993)^c^	India	Increased	OR = 7·07 (3·02, 16·7)^b^	Increased	OR = 9·17 (4·02, 21·1)^b^
McGready *et al.* (2004)^e^	Thailand	Similar	OR = 1·00 (0·01, 81·3)^b^	Increased	OR = 3·5 (0·46, 157·2)^b^
Tobon-Castano *et al.* (2011)	Colombia	Increased	RR = 1·47 (0·95, 2·28)	Increased	RR = 3·17 (2·02, 4·97)
Mean difference in gestational age					
Nosten *et al.* (1999)	Thailand	Similar	MD = 0 weeks (−0·3, 0·3)^b^	Similar	MD = −0·2 weeks (−0·4, 0.0)^b^
McGready *et al.* (2004)^e^	Thailand	Similar	MD = 0·5 weeks (−0·79, 1·79)^b^	Similar	MD = −0·4 weeks (−1·35, 0·55)^b^
Yasnot *et al.* (2013)	Colombia	Decreased	MD = −1·9 weeks (−3·11, −0·69)	N/A	N/A
Machado Filho *et al.* (2014)	Brazil	Decreased	MD = −2 weeks	N/A	N/A
Odds of miscarriage					
Nair and Nair (1993)^c^	India	Increased	OR = 4·64 (0·63, 52·4)^b^	Increased	OR = 20·4 (4·40, 187)^b^
Nosten *et al.* (1999)	Thailand	Decreased	OR = 0·65 (0·41, 0·97)^b^^,^^f^	N/A	N/A
McGready *et al.* (2012)	Thailand	Increased^g^	OR = 3·99 (3·10, 5·13)^h^^,^^i^ OR = 2·70 (2·04, 3·59)^h^^,^^j^	Increased^g^	OR = 3·99 (3·10, 5·13)^h^^,^^i^ OR = 2·70 (2·04, 3·59)^h^^,^^j^

NB – ratios within 0·2 of 1 were considered similar to 1. Birth weight MDs <50 g were considered similar. Gestational age MDs <1 week were considered similar. Low birth weight defined as <2500 g. All measures of association are unadjusted unless otherwise specified. Abbreviations: MD, mean difference; OR, odds ratio; RR, Risk Ratio.

a Cases included *P. malariae* and *P. vivax* cases.

b Calculated from data in paper.

c Women in study restricted to those with history of fever.

d Adjusted for age, location, gestational age at first visit, compliance to attendance at the antenatal clinic.

e Adjusted for hookworm infection, vitamin A deficiency and trimester of pregnancy.

f Error in the published paper, the reported events in the *P. vivax* group should read 447, not 44. Confirmed by authors of the paper.

g Single episode of *P. vivax* or *P. falciparum* in first trimester.

h Adjusted for age, smoking and estimated gestational age.

i Symptomatic malaria.

j Asymptomatic malaria.

It is well documented that the risk of *P. falciparum* infection during pregnancy is highest amongst primigravid women (reviewed in Desai *et al.* 2007). Studies in Thailand, India and Indonesia have also found that primigravidae are more at risk of *P. vivax* infection than multigravidae (Brabin *et al.* 1990; Singh *et al.* 1998, 1999; Nosten *et al.* 1999; Poespoprodjo *et al.* 2008; Fowkes *et al.* 2012), although this finding is not consistent across all study sites (Singh *et al.* 1995; Luxemburger *et al.* 2001; Appleyard *et al.* 2008) (Table 4). Broad immunological and hormonal changes that take place with successive pregnancies could play a role in the decreasing risk of both *P. falciparum* and *P. vivax* with gravidity (Vleugels *et al.* 1987, 1989; Riley *et al.* 1989; Bouyou-Akotet *et al.* 2004, 2005). Additionally, a degree of protective immunity is acquired to both species during pregnancy, which may play a stronger role in *P. falciparum* infections than *P. vivax* infections (reviewed below).

**Table 4.** Risk/odds of *P. vivax* infection in primigravidae compared to multigravidae and comparisons with *P. falciparum* risk in co-endemic areas

Study	Country	*Plasmodium vivax*	Magnitude (95% CI)	*Plasmodium falciparum*	Magnitude (95% CI)
Brabin *et al.* (1990)	Papua New Guinea	Increased^a^	IRR = 2·17 (0·74, 6·45)^b^	Increased	IRR = 1·26 (0·90, 1·75)^b^
Singh *et al.* (1995)^c^	India	Similar	OR = 0·88 (0·41, 1·88)^b^	Increased	OR = 2·44 (1·59, 3·75)^b^
Singh *et al.* (1998)	India	Increased	RR = 1·57 (0·83, 2·97)	Similar	RR = 0·98 (0·61, 1·60)
Nosten *et al.* (1999)	Thailand	Increased	OR = 1·63 (1·37, 2·06)^d^	N/A	N/A
Singh *et al.* (1999)^c^	India	Increased	OR = 1·28 (0·83, 1·96)^b^	Increased	OR = 1·54 (1·09, 2·13)^b^
Luxemburger *et al.* (2001)	Thailand	Similar	Data not shown	Increased	RR = 1·39 (1·13, 1·70)^b^
Singh *et al.* (2001)	India	Decreased	RR = 0·50 (0·18, 1·42)^b^	Decreased	RR = 0·80 (0·59, 1·07)^b^
Appleyard *et al.* (2008)	Solomon Islands	Similar	RR = 1·03 (0·11, 9·35)^b^	Increased	RR = 2·69 (1·08, 6·71)^b^
Poespoprodjo *et al.* (2008)	Indonesia	Increased	OR = 1·4 (1·0, 2·0)	Increased	OR = 1·4 (1·0, 1·8)
Fowkes *et al.* (2012)	Thailand	Increased	OR = 1·89 (1·04, 3·33)	Increased	OR = 1·72(0·97, 3·03)

NB – ratios within 0·2 of 1 were considered similar to 1. Abbreviations: OR, odds ratio; RR, risk ratio; IRR, incidence rate ratio; N/A, not available. All measures of association are unadjusted unless otherwise specified.

a *Plasmodium vivax* and *P. malariae.*

b Calculated from data in paper.

c Women in study restricted to those with history of fever.

d Adjusted for age, location, gestational age at first visit, compliance to attendance at the antenatal clinic.

A review of the epidemiological data indicates that consolidation of data is challenging due to differences in transmission and clinical criteria. In summary, the data suggest that pregnant women may be at an increased risk of *P. vivax* during pregnancy, but are relatively more susceptible to *P. falciparum* than *P. vivax* compared to their non-pregnant counterparts. Infection with *P. falciparum* during pregnancy tends to lead to more severe negative maternal and birth outcomes than infection with *P. vivax*. Evidence suggests that primigravidae are at increased risk of *P. falciparum* and *P. vivax* compared to multigravidae. The differential risk, severity and gravidity effects could be attributed to the distinct pathologies of *P. falciparum* and *P. vivax* during pregnancy and/or differential immunity to the two species.

### *Key differences in* P. falciparum *and* P. vivax *clinical pathogenesis*

During pregnancy, specific *P. falciparum* variants emerge that can escape pre-existing immunity and sequester in the placenta. *Plasmodium falciparum* isolates in pregnant women upregulate the expression of *Pf*VAR2CSA, an antigen located on the *P. falciparum*-infected erythrocyte (*Pf*-IE) surface. *Pf*VAR2CSA is a specific form of the variant protein PfEMP1 (*P. falciparum* erythrocyte membrane protein 1) that binds to placental chondroitin-sulphate A (CSA) and helps mediate parasite sequestration in the placenta (reviewed in Khunrae *et al.* 2010). The increased burden and detrimental effects of *P. falciparum* infection observed in pregnant women has been largely attributed to elevated parasite densities and the placental sequestration of *Pf*-IEs (reviewed in Desai *et al.* 2007; Hviid and Salanti, 2007; Rogerson, 2010; Umbers *et al.* 2011). *Plasmodium falciparum* infection during pregnancy is typically associated with a very pronounced sequestration, or selective accumulation, of mature forms of blood-stage parasites in the placenta with a parasitaemia many fold higher than that observed in the peripheral blood (Walter *et al.* 1982; Beeson *et al.* 2002). The accumulation of large numbers of *Pf*-IEs at the placenta results in changes to placental histology including inflammation, deposition of pigment in fibrin or inflammatory cells, syncytial knotting and thickening of the trophoblastic basement membrane (Walter *et al.* 1982; Bulmer *et al.* 1993; Ismail *et al.* 2000; Rogerson *et al.* 2003). *P. vivax* lacks the *Pf*VAR2CSA protein, or any known *Pf*VAR2CSA orthologues, and *P. vivax-*IEs (*Pv*-IEs) are rarely found in the placenta (Singh *et al.* 2003; Mayor *et al.* 2012*a*; Carmona-Fonseca *et al.* 2013). Despite this, infections with *P. vivax* during pregnancy have been associated with some of the same histological changes observed in *P. falciparum* infections, though these changes are typically less severe (McGready *et al.* 2004; Souza *et al.* 2013) (Table 5). The binding of *Pv*-IEs to CSA (as well as other endothelial cells) has been described *in vitro* and may be partly mediated by *Pv*VIR (Variant Interspersed Repeats) proteins expressed on the surface of *Pv-*IEs. However, the level of cytoadhesion of *Pv-*IEs to CSA is around ten-fold lower than that displayed by *Pf-*IEs (Carvalho *et al.* 2010; Chotivanich *et al.* 2012) and cytoadherence to CSA does not differ between *P. vivax* isolates from pregnant and non-pregnant individuals (Marin-Menendez *et al.* 2013). The low level of CSA-adherence exhibited by *Pv-*IEs likely plays a minor role in pathogenesis compared to *P. falciparum.* The existence of *Pf*VAR2CSA in *P. falciparum* represents a crucial difference between the two species and explains much of the different infection outcomes experienced by pregnant women. The reduced level of *P. vivax* cytoadhesion in *vitro* relative to *P. falciparum* explains the rarity of clinical observations of *P. vivax* placental sequestration (Mayor *et al.* 2012*a*; Carmona-Fonseca *et al.* 2013; Souza *et al.* 2013; Chaikitgosiyakul *et al.* 2014). Although other ligand/receptor combinations have been proposed as contributing to the sequestration of parasites in the placenta (Table 5) the evidence currently favours *Pf*VAR2CSA/CSA as the principal interaction responsible for placental sequestration of *P. falciparum*.

**Table 5.** Key differences in *P. falciparum* and *P. vivax* placental and binding pathogenesis

Feature	*Plasmodium vivax*		*Plasmodium falciparum*	
	Finding	References	Finding	References
Infections detected in placenta	Rare	Singh *et al.* (2003) Mayor *et al.* (2012*a*) Carmona-Fonseca *et al.* (2013)	Common	Singh *et al.* (2003)
Altered placental histology	Yes (less severe than *Pf*)	McGready *et al.* (2004) Souza *et al.* (2013)	Yes (more severe than *Pv*)	McGready *et al.* (2004) Souza *et al.* (2013)
Binding to placental cryosection *in vitro*	Yes (decreased binding compared to *Pf*)	Carvalho *et al.* (2010)	Yes (increased binding compared to *Pv*)	Carvalho *et al.* (2010)
Binding to Chondroitin Sulphate-A *in vitro*	Yes	Chotivanich *et al.* (2012)	Yes	Fried and Duffy (1996)
Binding to hyaluronic acid *in vitro*	Yes	Chotivanich *et al.* (2012)	Yes	Beeson *et al.* (2000)
Binding to IgG *in vitro*	Unknown	N/A	Yes	Flick *et al.* (2001)
Binding to IgM *in vitro*	Unknown	N/A	Yes	Creasey *et al.* (2003)
Differential binding in pregnancy compared to non-pregnancy isolates	No	Marin-Menendez *et al.* (2013)	Yes	Fried and Duffy (1996) Beeson *et al.* (1999)
Leading placental ligand candidate	Members of the VIR family	(Carvalho *et al.* 2010; Chotivanich *et al.* 2012)	*Pf*VAR2CSA	Salanti *et al.* (2003)
Rosetting	Yes	Udomsanpetch *et al.* (1995)	Yes (uncommon in placental isolates)	Handunnetti *et al.* (1989) Rogerson *et al.* (2000)

Abbreviations: *Pf, Plasmodium falciparum; Pv, Plasmodium vivax*; IgG, immunoglobulin G; IgM, immunoglobulin M; N/A, not available.

Another pathophysiological feature mediated by IE surface ligands is rosetting, whereby IEs bind to uninfected erythrocytes. Rosetting is a feature of both *P. falciparum* and *P. vivax* isolates from infections in non-pregnant individuals (Udeinya *et al.* 1981; Udomsanpetch *et al.* 1995). Recent evidence suggests that rosetting occurs more frequently in *P. vivax* isolates than *P. falciparum* isolates (Lee *et al.* 2014) likely due to differential erythrocyte or receptor preferences. Glycophorin C is a ligand for both *P. falciparum* and *P. vivax* rosetting (Lee *et al.* 2014) whilst numerous other receptors have been identified for *P. falciparum* rosetting (reviewed in Sherman *et al.* 2003). Interestingly, rosetting is uncommon in placental *P. falciparum* isolates (Maubert *et al.* 1998; Rogerson *et al.* 2000) and is lacking in isolates that adhere to CSA and upregulate VAR2CSA (Beeson and Brown, 2004). In the absence of placental sequestration, the rosetting phenotype may contribute more strongly to clinical outcomes in *P. vivax* infection; rosetting is strongly associated with anaemia and increased parasitaemia in both *P. falciparum* and *P. vivax* infection (Rowe *et al.* 2002; Doumbo *et al.* 2009; Marin-Menendez *et al.* 2013).

In the absence of considerable interactions between the placenta and *Pv*-IEs, the altered placental histology associated with *P. vivax* infection is likely due to broad effects of peripheral infection, such as maternal anaemia, fever or the effect of the cytokine response to infection (Mayor *et al.* 2012*a*; Souza *et al.* 2013). These mechanisms likely also act in *P. falciparum* peripheral infections in conjunction with the direct effects of placental sequestration. Reticulocytosis occurs in some populations during pregnancy and may contribute to an increased risk of *P. vivax* (Traill, 1975). Taken together, current data show that interactions between *P. vivax* and placental receptors are rare in contrast to the common interaction of *P. falciparum* ligands with placental receptors which largely explains the reduced magnitude of negative outcomes in *P. vivax* infections in pregnancy compared to *P. falciparum* infections.

### Plasmodium falciparum *and* P. vivax *risk in the postpartum period*

How the increased burden and risk of *P. falciparum* and *P. vivax* malaria during pregnancy relates to the risk of malaria in the postpartum period is the focus of increasing research. The World Health Organization defines the postpartum period as beginning 1 h after the delivery of the placenta and continuing until 6 weeks after the birth of the infant (WHO, 2011). This definition is rarely adhered to in the malaria literature, so for the purpose of this review the postpartum period is defined as the period from delivery to 6 months post-delivery. The majority of postpartum studies have been conducted in Africa and have compared the risk of *P. falciparum* infection postpartum to the risk during pregnancy; with these studies observing a reduced risk of *P. falciparum* infection in the postpartum period (Table 6) (Bray and Anderson, 1979; Watkinson and Rushton, 1983; Steketee *et al.* 1996; Fievet *et al.* 1997; Green *et al.* 2007; Menendez *et al.* 2008; Serra-Casas *et al.* 2011). Conversely, the single study that investigated *P. falciparum* clinical malaria found an increased risk during the 60 days postpartum compared to each trimester of pregnancy (Diagne *et al.* 2000). The sole study assessing the risk of *P. vivax* and *P. falciparum* infection in postpartum compared to pregnant controls (in Papua New Guinea) found the incidence of *P. vivax* and *P. falciparum* parasitaemia increased from delivery until 4 months postpartum (Brabin *et al.* 1990) with a greater relative increase in postpartum *P. vivax* incidence than *P. falciparum* incidence. Importantly, chemoprophylaxis was ceased at delivery so this finding may be more reflective of a ‘rebound effect’ than an indication of the natural course of susceptibility during the postpartum period (Brabin *et al.* 1990). Overall, the heightened risk of *P. falciparum* seen during pregnancy is typically reduced in the postpartum period, whilst the limited evidence of *P. vivax* risk postpartum compared to pregnancy suggests that the risk is elevated.

**Table 6.** Risk of *P. vivax* and *P. falciparum* in the postpartum period

Study	Country	Postpartum period	*Plasmodium vivax*	Magnitude (95% CI)	*Plasmodium falciparum*	Magnitude (95% CI)
Risk/odds of infection compared to pregnancy						
Bray and Anderson (1979)	The Gambia	At 1 mo.	N/A	N/A	Similar^a^	OR = 0·81 (0·41, 1·53)^b^
Brabin *et al.* (1990)	Papua New Guinea	Across 4 mos.	Increased^a^	IRR = 4·89 (1·92, 11·59)^b^	Increased^a^	IRR = 1·28 (0·78, 2·00)^b^
Steketee *et al.* (1996)	Malawi	At 2 mos.	N/A	N/A	Similar^a^	OR = 0·80 (0·72, 0·88)^b^
Fievet *et al.* (1997)	Cameroon	At 6 mos.	N/A	N/A	Decreased^a^^,^^c^	OR = 0·19 (0·05, 0·68)^b^
Diagne *et al.* (2000)	Senegal	Across 2 mos.	N/A	N/A	Increased^a^^,^^d^	IRR = 1·75 (0·75, 3·76)^b^
Green *et al.* (2007)	Kenya	At 2 mos.	N/A	N/A	Decreased^a^	OR = 0·20 (0·00, 1·80)^b^
Menendez *et al.* (2008)	Mozambique	At 2 mos.	N/A	N/A	Decreased^a^	OR = 0·36 (0·22, 0·58)^b^
Serra-Casas *et al.* (2011)	Mozambique	At 2 mos.	N/A	N/A	Decreased^e^	OR = 0·35 (0·19, 0·61)
Risk/odds of clinical malaria compared to pregnancy						
Diagne *et al.* (2000)	Senegal	Across 2 mos.	N/A	N/A	Increased^f^	IRR = 1·98 (0·84, 4·36)^b^
Risk/odds of infection compared to non-pregnant women						
Diagne *et al.* (2000)	Senegal	Across 2 mos.	N/A	N/A	Increased^a^^,^^d^	RR = 1·8 (1·1, 2·7)^g^
Ramharter *et al.* (2005)	Gabon	Across 2·5 mos.	N/A	N/A	Increased^a^	IRR = 2·7 (1·0, 8·2)
Boel *et al.* (2013)	Thailand	Across 3 mos.	Increased^a^	HR = 1·34 (1·05, 1·72)	Decreased^a^	HR = 0·39 (0·21, 0·72)
Risk/odds of clinical malaria compared to non-pregnant women						
Diagne *et al.* (2000)	Senegal	Across 2 mos.	N/A	N/A	Increased^f^	RR = 4·1 (1·8, 9·5)^g^
Ramharter *et al.* (2005)	Gabon	Across 2·5 mos.	N/A	N/A	Increased^h^	IRR = 9·8 (1·4, 420·0)

NB – ratios within 0·2 of 1 were considered similar to 1. All measures of association are unadjusted unless otherwise specified.

Abbreviations: mos., months; LM, light microscopy; OR, odds ratio; IRR, incidence rate ratio; RR, risk ratio; HR, hazard ratio.

a Detected by light microscopy.

b Calculated from data in the paper.

c Study restricted to primigravid women only.

d Asymptomatic infection.

e Detected by PCR.

f Clinical malaria defined as any case of fever or fever-related symptoms associated with a ratio of parasites to leucocytes that exceeds a pyrogenic threshold.

g Adjusted for exposure, parity, duration of residence in village and effects within study subjects.

h Clinical malaria defined as asexual *P. falciparum* parasitemia with **>**100 parasites μL^−1^ of blood, fever (current or within the previous 24 h), or presence of other symptoms associated with malaria.

To truly evaluate whether malaria risk returns to non-pregnant levels immediately after pregnancy, the postpartum risk needs to be compared to non-pregnant controls. Two studies in Africa (Senegal and Gabon) found an increased risk of *P. falciparum* infection relative to non-pregnant women (Diagne *et al.* 2000; Ramharter *et al.* 2005) (Table 6). Both studies also found a greater increase in the risk of clinical malaria relative to the risk of *Plasmodium* spp. infection indicating that the postpartum state is more susceptible to clinical malaria than the non-pregnant state independent of an increased risk of infection. Depressed immunity may explain this finding with the Senegal study finding that after 90 days postpartum the risk of clinical *P. falciparum* malaria returned to the level seen prior to pregnancy, suggesting that the responsible factor for altered postpartum risk returns to normal after 3 months (Diagne *et al.* 2000). In contrast to African findings, a recent study on the Thai–Myanmar border found that postpartum women had decreased risk of *P. falciparum* episodes than age and location matched non-pregnant controls, whilst there was an increased risk of *P. vivax* episodes in postpartum women compared to non-pregnant controls (Boel *et al.* 2013). Further research into postpartum risk of malaria will help address the current conflicting evidence of the risk of malaria in the postpartum period.

It has been suggested that the differential risk of malaria in the postpartum period may be due to immunological changes that occur during pregnancy and gradually return to ‘normal’ in the postpartum period (Diagne *et al.* 2000; Ramharter *et al.* 2005). Immunological changes during pregnancy include changes in cell-mediated and humoral immunity (Jamieson *et al.* 2006; Robinson and Klein, 2012), which would presumably differentially affect susceptibility to *P. falciparum* and *P. vivax* due to underlying species differences in immunopathology.

### Immunity to P. falciparum and P. vivax in pregnancy

A variety of immunological changes occur during pregnancy, with changes in the nature of cytokine responses, a general suppression of cell-mediated immunity and increased humoral immunity (reviewed in Jamieson *et al.* 2006; Robinson and Klein, 2012). This shift is believed to reduce the chance of foetal rejection and increase the maternal transfer of antibodies to the foetus. These broader immunological changes are also likely to contribute to an altered susceptibility to both *Plasmodium* spp. during pregnancy in addition to the availability of the placenta as a sequestration site.

The broadly suppressed cell-mediated immunological state that exists during pregnancy should dampen the effectiveness of T cells on both *P. falciparum* and *P. vivax*. However, few studies have investigated the impact of an altered cell-mediated response on *Plasmodium* spp. infection during pregnancy. CD8 T cell levels are higher in the inflammatory infiltrate in chronically *P. falciparum* infected placentas compared to uninfected placentae, past infections, acute infections and placentae from non-exposed women (Ordi *et al.* 2001). This observation was supported by another study which found greater frequencies of CD8 T cells producing IFN-alpha and TNF-alpha in *P. falciparum* infected placentae compared to uninfected placentae (Diouf *et al.* 2007). Less is known about the role of T cells at the placenta during *P. vivax* infections. An increased presence of mononuclear cells in the placenta was detected in instances of *P. vivax* and *P. falciparum* infections compared to no infection, with similar numbers of mononuclear cells in *P. vivax* and *P. falciparum* infections (Souza *et al.* 2013).

Another important impact an altered cell-mediated immune response may have in pregnancy is an altered ability to control liver stage infection and *P. vivax* relapse. Though direct human evidence is lacking, cell-mediated immunity is thought to be particularly important for pre-erythrocytic immunity on the basis of animal models (reviewed in Doll and Harty, 2014). However, what constitutes an adequate immune response against clinical relapses of *P. vivax* is unknown as is the risk of relapses during the altered immunological state of pregnancy.

The humoral immune response is a crucial component of naturally acquired immunity and antibody responses to both *P. falciparum* and *P. vivax* antigens are important biomarkers of exposure and protective immunity in meta-analyses of non-pregnant populations (Fowkes *et al.* 2010; Cutts *et al.* 2014). Numerous studies have demonstrated the important role of anti-*Pf*VAR2CSA humoral immunity in *P. falciparum* infections during pregnancy (reviewed in Duffy, 2007; Hviid and Salanti, 2007; Rogerson, 2010; Ataide *et al.* 2013). Antibodies reactive against recombinant *Pf*VAR2CSA, and to the surface of erythrocytes infected with *P. falciparum* placental isolates and *P. falciparum* lines selected by their adhesion to CSA increase with gravidity (reviewed in Ataide *et al.* 2013), indicating that immunity to *Pf*VAR2CSA is acquired or boosted progressively with successive pregnancies, and is associated with parasite clearance and reduced odds of placental infection (Guitard *et al.* 2008; Feng *et al.* 2009; Tutterrow *et al.* 2012*a*, *b*). There is currently no complementary evidence for *P. vivax*. The risk of *P. vivax* also tends to decline with gravidity (Table 4), rendering the existence of *P. vivax* antigens that are upregulated in pregnancy and important as immune targets a viable hypothesis. However, in the absence of pregnancy-specific *P. vivax* isolates or antigens, the gravidity effect may also be explained by a broader acquisition and boosting of immunity towards *Pv*-IEs and merozoite antigens during exposure to *P. vivax* during pregnancy or merely a reflection of immune acquisition with age. Additional mechanisms that influence susceptibility to both species during pregnancies are increased cortisol concentrations (Vleugels *et al.* 1989; Bouyou-Akotet *et al.* 2005) and reduced NK cell activity (Bouyou-Akotet *et al.* 2004) particularly in primigravid pregnancies.

There is little data available on antibodies to merozoite antigens during pregnancy (including orthologues expressed in both *P. falciparum* and *P. vivax*, e.g. AMA1, MSP1_19_). Pregnant women in endemic settings have typically acquired protective immunity to these antigens during childhood; this immunity would likely contribute to a protective effect in pregnancy by reducing parasitaemia, which would have a knock-on protective effect on the burden of placental infection. Evidence to support this is limited, however some studies have found *ad hoc* associations with particular merozoite antigens (e.g. *Pf*MSP1-19 and *Pf*AMA-1) with improved birth outcomes in women exposed to *P. falciparum* (Taylor *et al.* 2004; Mayor *et al.* 2013). Results must be interpreted with caution, given the lack of similar associations with other non-pregnancy specific antigens in the same studies (*Pf*CSP, *Pf*LSA1 *Pf*RESA, *Pf*DBLγ, *Pf*DBLα, *Pf*MSP1-19, *Pf*AMA1 *Pf*EBA175). Furthermore, *P. falciparum* merozoite responses (and *P. vivax* responses in co-endemic areas) are often highly correlated with *Pf*VAR2CSA (Fowkes *et al.* 2012) so associations observed can serve as a proxy for higher levels of other protective responses.

Alternatively, in the absence of *P. vivax*-specific mechanisms, the gravidity effect could be indirect. It has been hypothesized that relapses of *P. vivax* infections are triggered by fever, notably by other malaria infections (reviewed in Shanks and White, 2013). If this were the case then one would expect *P. falciparum* erythrocytic immunity (both cell-mediated and humoral), to indirectly protect against *P. vivax* relapse by protecting against febrile symptoms. This indirect mechanism could explain the decreased risk of *P. vivax* with increasing gravidity in co-endemic regions in the absence of more direct immunological mechanisms.

Cross-species immunity also provides an alternative explanation for the gravidity effect of *P. vivax*. There is little reliable human data on cross-species immunity. An experimental infection of a non-pregnant individual with *P. vivax* showed that antibodies induced by *P. vivax*, are capable of recognizing *P. falciparum* schizont extract and may be able to inhibit *P. falciparum* growth *in vitro* (Nagao *et al.* 2008). How this translates vice versa or in pregnancy is unknown but may explain the interaction in disease severity between the two species in Thailand where *P. vivax* infection reduced the severity and number of *P. falciparum* episodes during pregnancy (Luxemburger *et al.* 1997; Nosten *et al.* 1999). Evidence also suggests that high-density blood stage infections may be able to inhibit liver stage infections through an increase in hepcidin levels (reviewed in Portugal *et al.* 2011). Mechanisms of *Plasmodium* species-transcending immunity are poorly defined in humans and require further elucidation to determine their role in pregnancy and postpartum.

There is a relatively scarce amount of literature regarding the role of non-IgG antibodies in *P. falciparum* and *P. vivax* infection during pregnancy and postpartum which is not surprising given that IgG is considered to be the key immunoglobulin for naturally acquired immunity against malaria (Doolan *et al.* 2009). IgM is typically observed in the primary response to infection and numerous *P. falciparum* and *P. vivax* antigens elicit IgM responses (Cutts *et al.* 2014). IgM has been shown to bind non-specifically to *Pf*VAR2CSA a feature which may have evolved as an immune evasion mechanism (Creasey *et al.* 2003; Elliott *et al.* 2005; Rasti *et al.* 2006; Semblat *et al.* 2006; Barfod *et al.* 2011). The binding of IgM to *Pf*VAR2CSA has been shown to interfere with specific IgG recognition and opsonic phagocytosis of IEs infected with pregnancy-specific isolates ((Barfod *et al.* 2011) but not other non-pregnancy specific *Pf*EMP-1s (Stevenson *et al.* 2014). IgM has also been implicated in rosetting and strengthening *Pf*-IE erythrocyte interactions (Stevenson *et al.* 2014) however rosetting is rare in *P. falciparum* placental isolates (Maubert *et al.* 1998; Rogerson *et al.* 2000). There is currently no data on the role of IgM in *P. vivax* rosetting. Further investigation of the role of IgM in *P. falciparum* and *P. vivax* infections is warranted.

The functional roles IgG antibodies require for protection against *P. falciparum* and *P. vivax* infection are fairly poorly defined. The predominant isotypes found against *P. falciparum* placental isolates are IgG1 and IgG3, the dominant isotypes against most malarial antigens (Elliott *et al.* 2005; Megnekou *et al.* 2005; Stanisic *et al.* 2009; Richards *et al.* 2010), which can function through adhesion-inhibition/invasion-inhibition, phagocytosis, antibody-dependent cell-mediated cytotoxicity and/or complement fixation. Anti-*Pf*VAR2CSA IgG can inhibit adhesion by interfering with the binding of *Pf*-IEs and CSA or recombinant *Pf*VAR2CSA to CSA (Ricke *et al.* 2000; Barfod *et al.* 2010; Khunrae *et al.* 2010). Opsonic phagocytosis against CSA-binding parasite isolates have been identified in sera from pregnant women (Keen *et al.* 2007; Tippett *et al.* 2007; Feng *et al.* 2009; Ataide *et al.* 2010, 2011; Barfod *et al.* 2010). There is little information at present on the contribution of anti-*Pf*VAR2CSA IgG to antibody-mediated complement activity, with some indications that excessive innate complement binding is detrimental (Conroy *et al.* 2009, 2013; Khattab *et al.* 2013). Antibody-mediated immune functions against a range of *P. falciparum* targets are present during pregnancy (Teo *et al.* 2014), but whether altered immunology during pregnancy alters their magnitude as compared to non-pregnant individuals is unknown. Studies on *Pv*-IE are severely hindered by the inability to culture *P. vivax* long-term *in vivo*.

In non-pregnant populations, clinical immunity is thought to develop more rapidly to *P. vivax* than *P. falciparum* as indicated from parasitological data from syphilis malariotherapy patients (Collins and Jeffery, 1999*a*; Collins *et al.* 2004) and from malaria endemic areas whereby the prevalence of *P. vivax* infection and clinical episodes peaks at younger ages compared to *P. falciparum* (Maitland *et al.* 1996; Smith *et al.* 2001; Mueller *et al.* 2009; Lin *et al.* 2010). It is hypothesized that this is due to a reduced immune threshold required to achieve protection against *P. vivax* compared to *P. falciparum* or the ability of *P. vivax* to relapse giving rise to a higher molecular force of infection (Koepfli *et al.* 2013). Species-specific differences in the rate of immune acquisition have yet to be reconciled in pregnancy but longitudinal studies show that antibody responses to both *P. falciparum* and *P. vivax* antigens during pregnancy are similarly dynamic in response to species-specific *Plasmodium* spp. exposure (Aitken *et al.* 2010; Fowkes *et al.* 2012; Ampomah *et al.* 2014*b*), lending support to the notion that regular exposure is required to maintain malarial immunity. Interestingly, a recent longitudinal study of antibodies in pregnancy found that antibodies to *P. vivax* (*Pv*AMA1) were not boosted with successive infections in pregnancy, in contrast with *P. falciparum* antibodies which were boosted with each exposure (including the homologue *Pf*AMA1) (Fowkes *et al.* 2012). This may indicate a difference in immunological memory or recall response between the two species or the much lower parasitaemia densities in *P. vivax* infections are less efficient in boosting responses. The implications of this lack of boosting for immunity and increased risk of *P. vivax* in pregnancy and the postpartum period (observed in the same study area) are unknown and further studies are necessary. Furthermore an understanding of antibody dynamics postpartum would help elucidate how pregnancy-favoured antibodies are maintained in between pregnancies with apparent limited exposure to pregnancy-favoured antigens.

### Antibody responses postpartum and between pregnancies

The strong link between gravidity and *Pf*VAR2CSA antibodies suggests that antibody responses and immune memory are maintained between pregnancies and postpartum when exposure to *Pf*VAR2CSA is low. This is at odds with the paradigm that frequent exposure is required to develop a long lasting antibody response to malaria and that, in the absence of repeated exposure, immunity is short lived (i.e. weeks) (Kinyanjui *et al.* 2003; Langhorne *et al.* 2008).

Antibodies are secreted by plasma cells, which can be either short-lived or long-lived (Manz *et al.* 2005). Mathematical modelling has demonstrated that separate populations of long and short lived cells can describe the rapid decay of antibodies observed immediately following exposure and the long-lived maintenance of a lower level of antibodies in African children (White *et al.* 2014). This is reflected in studies that have investigated antibody longevity. Estimates in individuals’ shortly after a drug treated acute episode of malaria typically find short *P. vivax* and *P. falciparum* antibody half-lives (6 to 52 days) (Soares *et al.* 1999; Kinyanjui *et al.* 2007) whereas studies investigating long-term decay of antibodies in uninfected individuals have estimated longer *P. vivax* and *P. falciparum* antibody half-lives in excess of 5 years (Drakeley *et al.* 2005; Wipasa *et al.* 2010). Additionally, antibodies have been detected in individuals who have not been exposed to either species in over 5 years (Luby *et al.* 1967; Druilhe *et al.* 1986; Braga *et al.* 1998; Wipasa *et al.* 2010; Moncunill *et al.* 2013; Ndungu *et al.* 2013).

Little is known about antibody longevity in pregnancy and postpartum. A study in a low transmission co-endemic area of Thailand found that *P. vivax* and *P. falciparum* merozoite antibody response half-lives calculated during pregnancy were shorter than that calculated for *Pf*VAR2CSA responses and was longer in those who had been exposed (0·8–7·6 years for merozoite antigens *vs* 57·6–142 years for VAR2CSA (Fowkes *et al.* 2012)). While these estimates should not be directly extrapolated from pregnancy into the postpartum period, recent evidence from cohorts of pregnant and postpartum women provide further evidence for long-term antibody maintenance postpartum. A study in Mozambique found that women 1–2 months postpartum had a median level of antibodies against the surface of a placental parasite line (CS2) comparable (3·3% higher) to women at delivery (Mayor *et al.* 2012*b*). A study in Malawi found that at 6 months postpartum 72·3% of women were still seropositive for antibodies to CS2 surface antigens (Aitken *et al.* 2010). More than 40% of women in Ghana who had not been pregnant in 1–6 years remained seropositive to *Pf*VAR2CSA suggesting that there is some level of antibody response maintenance in the relative absence of exposure (Ampomah *et al.* 2014*a*, *b*). Importantly they also demonstrated that the level of *Pf*VAR2CSA specific IgG-secreting B cells did not depend on time since last pregnancy or number of pregnancies suggesting that *Pf*VAR2CSA B cell memory is stably maintained in the absence of exposure (Ampomah *et al.* 2014*a*).

Explanations for an apparent increased longevity of *Pf*VAR2CSA responses are unclear, but could relate to a large sequestered parasite load providing a strong and sustained antigenic stimulus or be reflective of boosting as a result of undetected placental infection during pregnancy, or the greater immune longevity that appears to occur in adults. It is thought that there is limited or infrequent exposure to *Pf*VAR2CSA prior to the first pregnancy, in contrast to most malarial antigens, which are generally encountered throughout life. However, studies have shown that antibodies to *Pf*VAR2CSA can be acquired in childhood (Beeson *et al.* 2007). This would influence subsequent response to *Pf*VAR2CSA in pregnancy such that antibody levels may be boosted more rapidly upon re-exposure and be better maintained. Younger individuals tend to have shorter half-lives than older individuals (Taylor *et al.* 1996; Akpogheneta *et al.* 2008) and the age of primary exposure to an antigen may affect the longevity of immune responses to that antigen. The detection of *Pf*VAR2CSA antibodies in women who have not been pregnant in years and the observed persistence of *Pf*VAR2CSA specific IgG-secreting B cells supports the hypothesis that *Pf*VAR2CSA antibodies acquired in earlier pregnancies are maintained to protect subsequent pregnancies against *P. falciparum.* However, further longitudinal studies of women followed after pregnancy are required to assess this.

Whether there are *P. vivax* antigens that are specifically upregulated in pregnancy and whether antibodies against *P.vivax* are maintained postpartum and throughout pregnancies is unknown. Furthermore, the extent to which the immunological changes that occur during pregnancy and postpartum influence the risk of *P. vivax* relapse is unclear. If cell-mediated immunity is important in controlling liver infection, as it is in mouse models (reviewed in Doll and Harty, 2014), then the dampening of cell-mediated immunity would have a greater impact on *P. vivax* than *P. falciparum* due to the former parasites relatively longer period of residence in the liver. More immunological research is needed to further understand how immunity relates to the differential risk of *P. falciparum* and *P. vivax* postpartum.

Box I. Research priorities

• Further epidemiological studies on the risk of *P. vivax* by pregnancy status and gravidity in different populations.
• Elucidate the mechanisms by which *P. vivax* infection during pregnancy contributes to negative maternal and infant outcomes.
• Quantify the clinical relevance of putative *in vivo P. vivax* binding to the placenta.
• Conduct longitudinal studies in pregnant women that incorporate humoral, cellular and functional immunity against both *P. vivax* and *P. falciparum* to quantify their relative contributions towards protection against infection and its course.
• Determine the risk of *P. vivax* and *P. falciparum* postpartum in different settings, ideally with both pregnant and non-pregnant comparison groups.
• Elucidate the immunological mechanisms of altered risk postpartum.
• Investigate the modulating effect of pregnancy on cell-mediated immunity in a malaria context.
• Discover the mechanisms that underpin the cause of *P. vivax* relapse.
• Identify immune correlates of protection against *P. vivax* relapse.
• Determine the contribution of cross-species immunity in naturally exposed human populations.

## FUTURE DIRECTIONS

Immunological evidence has helped provide a convincing explanation for the unique epidemiology of *P. falciparum* in pregnancy. Many questions remain to be answered in relation to *P. vivax* during pregnancy and the risk of both species postpartum (Table 7 and Box 1). Currently the availability of both immunological and epidemiological evidence pertaining to *P. vivax* in pregnancy is limited and inconsistent. A more comprehensive understanding of the epidemiology of *P. vivax* in pregnancy will act as a primer for future studies on the immunology of *P. vivax* in pregnancy. Ideally, comprehensive longitudinal studies that incorporate measurements of multiple immunological mechanisms would be able to assess the relative contribution of each of these functions towards protection and the observed epidemiology. Whether delivery marks the end of a period of increased risk of malaria is debatable. The epidemiology of the postpartum period remains unclear, with the few studies conducted providing conflicting results. Further epidemiological studies are needed to explore the differential risk of *P. falciparum* and *P. vivax* in the postpartum period, preferably in tandem with immunological studies, which may be able to explain the mechanisms underlying the epidemiology.

**Table 7.** Epidemiological observations of *P. falciparum* and *P. vivax* during pregnancy and postpartum and proposed mechanisms

Proposed species-transcending mechanisms	References	Proposed species-specific mechanisms	References
Increased parasitaemia/risk of infection during pregnancy – greater increase in *P. falciparum* than *P. vivax*			
Imunomodulation in pregnancy alters susceptibility	Roberts *et al.* (1996)	*Plasmodium falciparum* – placental sequestration	McGregor (1984)
Altered hormonal profiles during pregnancy	Bouyou-Akotet *et al.* (2005)	*Plasmodium vivax* – placental sequestration, though evidence is minimal	(Carvalho *et al.* 2010; Chotivanich *et al.* 2012)
Altered attractiveness to mosquitoes	Lindsay *et al.* (2000)	*Plasmodium vivax* – altered immunity/hormones in pregnancy alters risk of relapse	Roberts *et al.* (1996)
Reduced risk of infection with increasing gravidity			
Acquisition of overall immunity acquired with age	Doolan *et al.* (2009)	*Plasmodium falciparum* – humoral immunity to *Pf*VAR2CSA acquired with gravidity	Fried *et al.* (1998)
Altered hormonal profiles with gravidity	Bouyou-Akotet *et al.* (2005)	*Plasmodium vivax* – altered immunity/hormones with gravidity alters risk of relapse	Riley *et al.* (1989) Roberts *et al.* (1996)
Altered immunomodulation with gravidity	Riley *et al.* (1989)		
Altered iron deficiency with gravidity	Ouedraogo *et al.* (2012)		
Infection with both species leads to adverse birth outcomes, more severe in *P. falciparum* than *P. vivax*			
Systemic immune response to infection contributes to foetal growth restriction.	Umbers *et al.* (2011)	*Plasmodium falciparum* – sequestration at the placenta, leads to local pathology and immune response at the placenta	Umbers *et al.* (2011)
Anaemia	Friedman *et al.* (2009)		
Altered risk postpartum			
Behavioural changes postpartum alter level of exposure to vectors	Boel *et al.* (2013)	*P. falciparum* – increased susceptibility during pregnancy leads to increased immunity postpartum	This review
Hormonal/immunological profile in transition from pregnancy to ‘normal’	Diagne *et al.* (2000)		
